# Process Monitoring Using Synchronized Path Infrared Thermography in PBF-LB/M

**DOI:** 10.3390/s22165943

**Published:** 2022-08-09

**Authors:** Dennis Höfflin, Christian Sauer, Andreas Schiffler, Jürgen Hartmann

**Affiliations:** 1 Institute of Digital Engineering, University of Applied Sciences Würzburg Schweinfurt, 97070 Würzburg, Germany; 2 Bavarian Center of Applied Energy Research e.V., 97074 Würzburg, Germany

**Keywords:** SPIT, PBF-LB/M, additive manufacturing, process monitoring, SWIR, melt pool, galvanometer scanner

## Abstract

Additive manufacturing processes, particularly Laser-Based Powder Bed Fusion of Metals (PBF-LB/M), enable the development of new application possibilities due to their manufacturing-specific freedom of design. These new fields of application require a high degree of component quality, especially in safety-relevant areas. This is currently ensured primarily via a considerable amount of downstream quality control. Suitable process monitoring systems promise to reduce this effort drastically. This paper introduces a novel monitoring method in order to gain process-specific thermal information during the manufacturing process. The Synchronized Path Infrared Thermography (SPIT) method is based on two synchronized galvanometer scanners allowing high-speed and high-resolution observations of the melt pool in the SWIR range. One scanner is used to steer the laser over the building platform, while the second scanner guides the field of view of an IR camera. With this setup, the melting process is observed at different laser powers, scan speeds and at different locations with respect to the laser position, in order to demonstrate the positioning accuracy of the system and to initially gain thermal process data of the melt pool and the heat-affected zone. Therefore, the SPIT system shows a speed independent overall accuracy of ±2 Pixel within the evaluated range. The system further allows detailed thermal observation of the melt pool and the surrounding heat-affected zone.

## 1. Introduction

**Figure 1:**
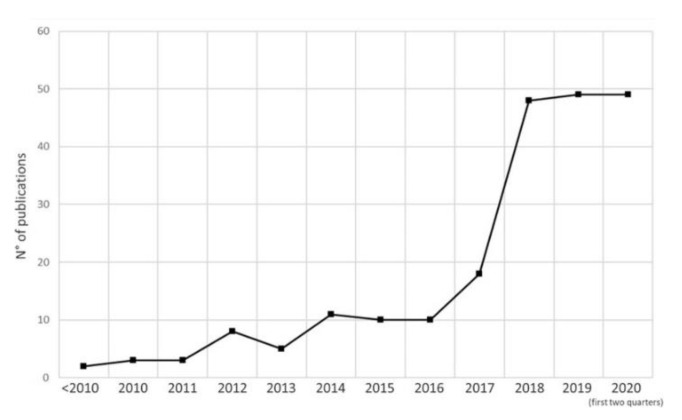
Number of publications on the topic of in situ sensing and/or in situ monitoring of powder bed fusion processes [3].

Since the earliest days of invention, the tremendous potential of metal processing additive manufacturing technologies has excited many researchers and industrial communities. The PBF-LB/M process, which is currently the most widespread technology with the highest degree of industrial maturity, plays a key role [1]. This technology enables the production of material- and weight-efficient innovative components without additional tools or clamping devices. However, the layer-wise melting of metal powder via laser heating results in complex and time-dependent temperature profiles that decisively depend on process and material parameters. The application of high scan velocities results in exposure times in the range of milliseconds and, in combination with high laser powers, induces extremely high heating and cooling rates, causing unique material properties and microstructures [2]. The control of these extreme conditions, with the aim of ensuring the required component quality, is still the major challenge of this technique. The immense increase in published research studies on this topic in recent years (cf. Figure 1) shows that the development is not yet complete, and there is still a need for dedicated research, especially in the area of in situ process data acquisition and validated in situ process monitoring.

**Figure 2:**
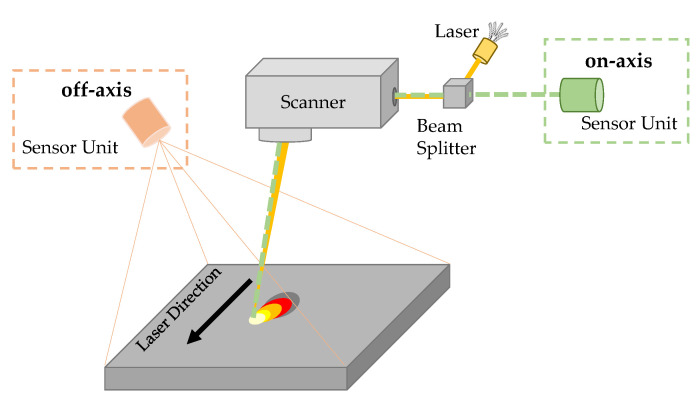
Schematic representation of the differently arranged sensor units of on-axis and off-axis systems.

Numerous in situ monitoring approaches for specific process parameters and characteristics have been investigated, with the aim of deriving indicators or key amounts for the subsequent evaluation of process and component quality. Among other things, laser power [4], powder coating and powder bed surface [5], powder bed compaction [6], smoke plumes and spatter behavior [7], particle emissions [8], part distortion [9] and vibration during powder coating [10] were already considered. However, the main focus of research efforts in the field of in situ process monitoring is in the investigation of the spatial and temporal temperature distribution in and around the melt pool, i.e., the laser–material interaction zone [11,12]. The systems used for this purpose are mainly designed for measuring thermal radiation, and are based on non-contact measurement devices such as diodes, pyrometers and cameras sensitive at different wavelength ranges [13,14,15]. Based on the arrangement of the optical path, these systems can be divided into off-axis and on-axis systems (cf. Figure 2) [16]. 

In off-axis systems, the field of view of the sensor unit is arranged at a fixed angle to the working plane, independently of the processing optics. This allows measurements in broad wavelength ranges within the visible [17,18] as well as the IR range (SWIR [19], MWIR [20], LWIR [21]). Off-axis monitoring systems of commercial PBF-LB/M systems, in which the whole processing area is to be considered, focus either on a high recording speed or a high spatial resolution due to limited hardware properties [22,23]. Zhang presented an investigation based on an off-axis setup with a limited viewing area of 12 mm × 12 mm, using high frame rate and high spatial resolution [7]. Emitted radiant intensity was measured to investigate various process-specific features as indicators for evaluating manufacturing quality. The single-track scenarios were detected with an image acquisition rate of 2000 fps and spatial resolution of 12 µm per pixel, in a wavelength range from 350 nm to 800 nm.

In on-axis systems, thermal process emissions are directed through the optical path of the laser onto the sensor unit. The field of view follows the beam of the laser over the entire processing surface, which is why these systems are particularly suitable for observing and characterizing the region in and around the melt pool. The small measuring field allows high image acquisition rates and high spatial resolution, with no limitations in the overall viewing area. To avoid imaging errors caused by the optical path, the observation wavelength is in the range of the wavelength of the processing laser [24]. Using an on-axis setup consisting of a high-speed camera and a photodiode, Berumen et al. [25] succeeded in capturing the melt pool during processing, with a resolution of 10 µm per pixel and an acquisition rate of 16.666 fps. The camera observed the melt pool dimensions, while the diode detected the average emitted thermal radiation. Based on the quantitative measurement data, a closed-loop control system was implemented to stabilize the melt pool. Clijsters et al. [26] adopted a similar arrangement, which is under development for real-time monitoring and detection of when melt breakage or material discontinuity occurs in the process.

Due to the complexity of the processes involved in PBF-LB/M, alongside the fact that individual sensors are limited to detect specific characteristics only, multi-sensor systems have been used more frequently to increase the detection quality. Harbig et al. [27] integrated an additional on-axis high-speed camera (HSC1; plasmoEye) into the existing melt pool monitoring system of an EOS M290, in order to detect defects based on process anomalies. The used melt pool monitoring system consisted of an on-axis and an off-axis photodiode, collecting the intensity data of the process in a range from 400 nm to 900 nm, while a high-speed camera provided a spatially resolved 2D intensity distribution of the melt pool at a wavelength of 900 nm ± 50 nm. Using a new methodology for the data fusion enabled a significant increase in the sensitivity of defect detection by up to 20%. 

In order to achieve a sufficiently high signal, especially for narrow-wavelength band measurements, the selected sensitivity range of the process monitoring system for thermal radiation measurement is important.

Therefore, the spectral characteristics of the material under investigation are of great interest. Here, the emissivity is probably the most important factor to be determined. Emissivity is the ratio of the thermal radiation an object emits compared to that of a perfect black body at the same temperature (a black body is defined by reflectance *ρ* = 0, transmittance *τ* = 0 and absorptance *α* = 1) [28].

With known emissivity, the radiance of a real body can be determined via Planck’s law, given in Equation (1). It describes the emitted thermal power per wavelength and area, represented by the radiance *L_b,λ_* of a black body, as a function of temperature *T* and wavelength *λ*.

(1) $$L_{b,\lambda }\left(\lambda ,T\right)=\frac{2hc^{2}}{\lambda^{5}}{\left(exp\left(\frac{hc}{\lambda kT}\right)-1\right)}^{-1}$$

Here, *h* is the Planck’s constant, *c* is the speed of light and *k* is the Boltzmann constant. Wien´s displacement law further shows that the wavelength *λ_max_*, at which the radiation power has its maximum value in the black-body spectrum, displaces to the side of the shorter wavelengths with increasing temperature *T* [29].

(2) $$\lambda_{max}\;T=2897\;µm\;K$$

**Figure 3:**
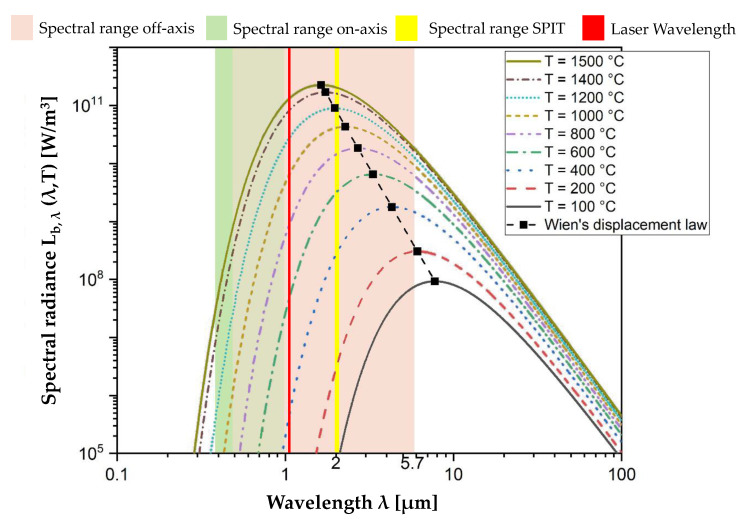
Visual representation of the spectral radiance distribution of a black body (Planck spectrum) and Wien’s displacement law for temperatures between 100 °C and 1500 °C [20]. The common spectral range of on-axis (light green area) and off-axis (light red area) systems, as well as the spectral range of the SPIT system (yellow bar) and the laser wavelength (red bar) are highlighted. The melting range of the material used in this investigation (AlMg3) is between 595 °C and 645 °C [30].

Figure 3 shows the spectral radiance distribution of a black body according to Planck, as well as the maximum wavelengths according to Wien for temperatures between 100 °C and 1500 °C. The picture depicts that the most relevant temperature regions of solidified surface temperatures have their maximum intensity in short- and mid-wavelength infrared (SWIR, MWIR). 

In this work, a novel system for in situ process monitoring is presented (Synchronized Path Infrared Thermography―SPIT) based on two separated Galvanometer scanners. With this setup, it is possible to reliably detect a roaming melt pool with a sensor unit whose movement can be controlled independently of the laser. The additional optical path is designed for wavelength ranges favorable for process monitoring in additive manufacturing (SWIR).

## 2. Materials and Methods

### 2.1. SPIT Setup

The experimental setup was designed to represent the basic processes taking place in the PBF-LB/M process (components are given only for clarity, which does not mean the used component is the best or the only applicable one). A Yb-fiber laser from JPT (JPT Opto-electronics Co., Shenzhen, China) operating at a wavelength of 1080 nm was used as the energy source. The laser output can be adjusted in the range from net 61 W to 458 W, as continuous wave or pulsed, with a frequency of up to 20 kHz. After being collimated and widened, the laser was guided into a SCANLAB intelliSCAN III 20 galvanometer scanner (SCANLAB GmbH, Puchheim, Germany)―the so-called laser scanner. The scanner directed the emitted radiation through an F-θ-Lens optimized for a wavelength range from 1030 nm to 1080 nm.

The lens focused the laser beam onto a platform placed 421 mm below in a sealed aluminum containment box. The approximated spot size at the focal point had a diameter of 65 μm, with a Rayleigh length of 1.5 mm. The laser and scanner unit could be controlled using a laserDESK software interface. The platform itself was movable along the *Z*-axis to adjust the different thicknesses of the processed parts. To control the status inside the sealed box, an additional diode was used to monitor the radiation emission occurring while the laser was activated.

**Figure 4:**
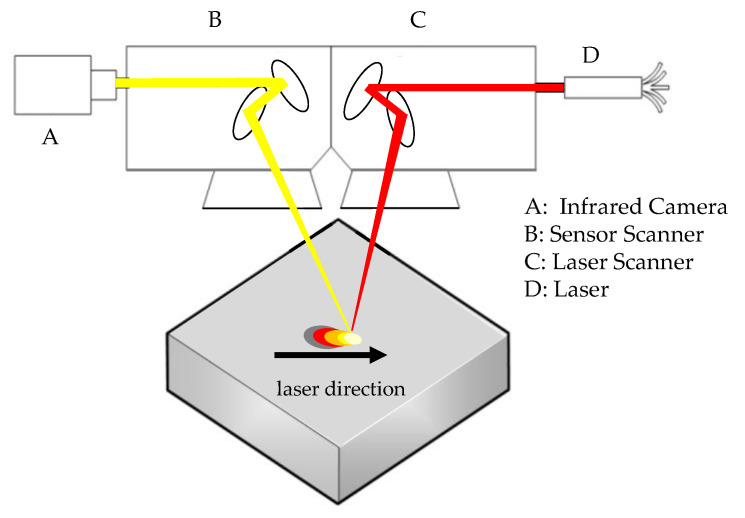
Schematic setup of Synchronized Path Infrared Thermography (SPIT). The measuring field of the camera (**A**) is moved by the sensor galvanometer scanner (**B**) synchronously with the working galvanometer scanner (**C**) of the laser (**D**).

To make the measuring point independent from the position of the processing laser, the SPIT setup was fitted with a second scanner unit―the so-called sensor scanner. A schematic representation of the setup is shown in Figure 4. The additional sensor scanner is, in principle, equivalent to the laser scanner, but with its optical components optimized for wavelengths in the SWIR range (1940 nm to 2050 nm). To adjust the slightly longer focal distance, an aluminum plate with a thickness of 16 mm was placed underneath the sensor scanner. This sets up a second optical path which is controlled by the same software interface as the main scanner. To superimpose the zero point of the coordinate systems of the two scanners, a basic offset was added, respectively subtracted from the X-axes. Within the maximum operating parameters of the scanner units, the arrangement resulted in an intersection area of 125 × 218 mm^2^. Additionally, ScanLab provided correction parameters for each F-θ-Lens regarding the different refraction angles between the center and the outer zones. These datasets increased the precision of the positioning significantly, and were implemented as correction factors.

To gain infrared thermal radiation information about the melting process, an InfraTec IR 8300 camera (InfraTec GmbH, Dresden, Germany) was placed in front of the aperture of the sensor scanner. The camera itself used a 50 mm lens, resulting in an optical resolution of the complete path of 135 μm for each pixel. Since the setup allows the measuring field of the camera to be moved synchronously to the laser, even a small image section is sufficient to fully capture the region of interest during the process. To maximize the possible frame rate, the field of view was therefore set to (40·40) pixels in the center of the image. This corresponded to an area of about (5.5·5.5) mm^2^. With this configuration, including an integration time of 89 µs, the camera provided a framerate of 2000 fps. The extraction of the raw data was realized via a customized interface based on the Software Development Kit (SDK) provided by InfraTec. After defining a region of interest, the software surveilled this area for temperatures above a certain threshold and recorded a predefined number of pictures when triggered. The integration time of the camera system and the adjustment of the spectral sensitivity was configured by the SDK as well. The information provided within the processed images shows the currently uncalibrated grayscale values of the laser interaction zone and the surrounding area.

**Figure 5:**
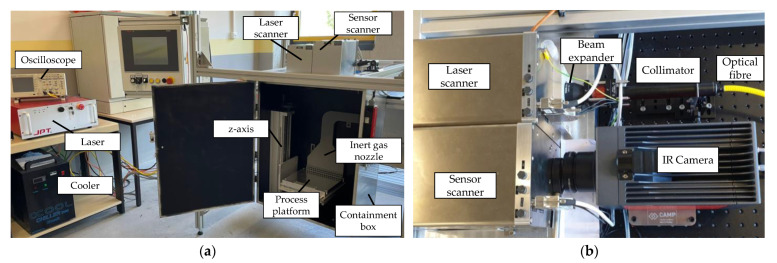
(**a**,**b**) Representation of the laboratory setup for the Synchronized Path Infrared Thermography system.

For downstream data analysis, the collected data were also stored as comma-separated value files that were analyzed and visualized with the help of MATLAB R2021a. Assuming that the brightest pixel always represents the laser–material interaction zone, the detection of this pixel can be used to draw conclusions about the positioning accuracy of the sensor system. Therefore, statistics about the position of the brightest pixel were carried out. Pictures taken during the time when the laser was deactivated, e.g., during jumps between separated characters, were not considered. The entire setup is shown in Figure 5.

### 2.2. Methods, Samples and Test Parameters

To initially evaluate the performance of the laser source and optical path, a series of tests were performed. The laser power was measured by a Primes Cube M, the beam caustic and the focus diameter with the help of a Primes FMW+ and, finally, the precision of the scan field with a Primes SFM (PRIMES GmbH, Max-Planck-Str. 2, 64319 Pfungstadt, Germany).

**Figure 6:**
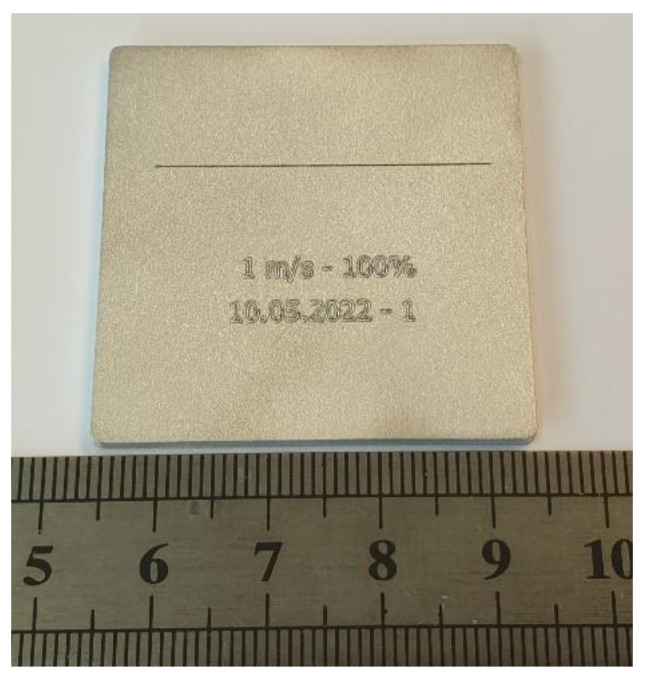
Processed AlMg3 base plate.

To further validate the accuracy of the setup and the ability to detect the thermal effects of laser–material interaction, a series of sample exposures were performed at different laser speeds and laser powers on plain aluminum plates. The parameter range is shown in Table 1. For every combination of laser speed and laser power, the path consisted of a straight line along the *X*-axis with a length of 32 mm. After completion of the line, the current test parameters and date were engraved (cf. Figure 6). The data produced while engraving the current parameter set and date were also part of the evaluation in order to represent complex laser paths in the x–y plane. Therefore, a font size of approximately 2.5 mm was chosen. Sandblasted plates made of AlMg3 with the dimensions of 40 × 40 mm^2^ and a thickness of 2 mm were used as the base material.

The investigation of the melt pool dimensions was carried out metallographically. For this purpose, the samples were cut perpendicular to the scan direction of the line, embedded in epoxy, ground, polished and etched. The microscopic investigation was performed using an incident light microscope (Olympus AX70).

## 3. Results

In the first step, the characteristics of the laser beam were determined. It proved to be well within the expected parameters, with a spot diameter of 66 μm, a focal length of 427 mm and a Rayleigh length of 1.43 mm.

**Figure 7:**
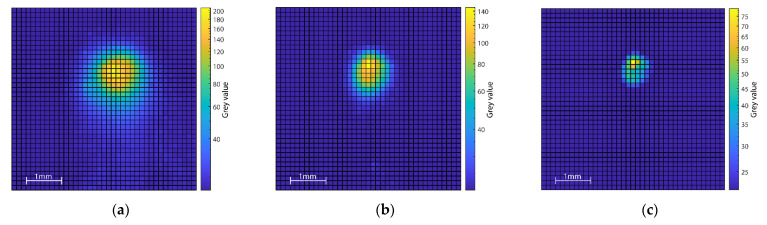
IR-images for (**a**) 460 W, 0.05 m/s; (**b**) 240 W, 0,5 m/s; (**c**) 105 W, 1 m/s.

Based on static pointwise measurements at different locations in and near the zero point of the superimposed coordinate systems, the laser spot could be assigned to a defined single pixel at the sensor unit. Further, the evaluation of the distinctive thermal images of these measurements showed the single brightest pixel at the place where the beam interacts with the surface. Surrounding pixels also showed significantly increased gray values, representing the zone of thermal influence (cf. Figure 7). During initial dynamic measurements near the zero point, the brightest pixel of the measurements corresponded with the previously determined laser spot location on the sensor. Therefore, the brightest pixel in each image was used as the reference point for the precision evaluation of the setup.

The dimensions and intensity of the measured brightness depended on the chosen laser power and scan speed. The signal-to-noise ratio variated from 20.3 dB at 460 W and 0.05 m/s to 8.1 dB at 105 W and 1 m/s.

The observation of the brightest pixel showed that tracking the laser–material interaction point is possible with high precision within the parameters applied in this study. The migration of this pixel was minimal in the tests performed and showed a direct dependency on the position within the processing area. The high repeatability revealed that the causes of this movement are to be found in an optical distortion of the thermographic path.

**Figure 8:**
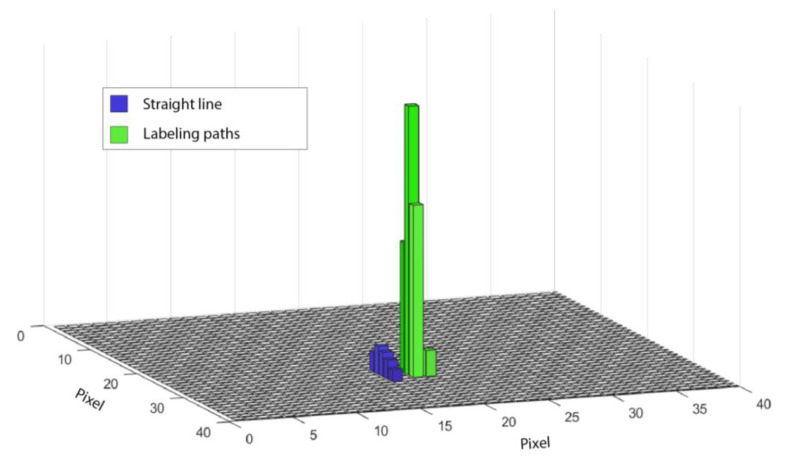
Frequency distribution of the single brightest pixel on the sensor at a laser power of 460 W and a scan speed of 1 m/s.

While moving along the x-axis to carry out the straight line, no deviation in the brightest pixel along the y-axis was observed. Meanwhile, the roaming within the field of view along the *x*-axis was consistent in all sets with a span of 5 pixels. After finishing the straight line, there was a jump along the y-axis to the beginning of the first digit. This jump can also be correlated to the omission of a whole senor line when analyzing the position of the brightest pixels. This skipped line is recognizable across all settings and, again, indicates the geometric relation between position and deviation (cf. Figure 8).

**Figure 9:**
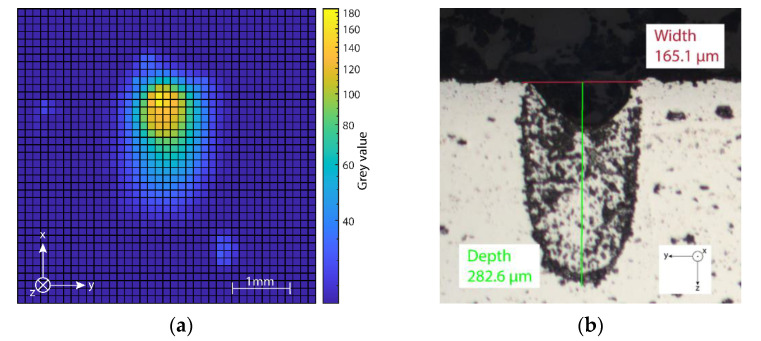
Comparison between: (**a**) A thermal image of an exposure with a laser power of 460 W and a scan speed of 1 m/s and (**b**) the metallurgical microscopy of the aluminum plate. The melt pool has a measured width of 165.1 μm and a measured depth of 282.6 μm.

In addition to the brightest pixel, a heat-affected zone can be observed in the thermography images (cf. Figure 9a), the dimension of which depended on the energy input into the surface, varying from approximately 2.2 mm at 460 W laser power and 0.05 m/s scan speed to 0.8 mm at 105 W laser power and 1 m/s scan speed. Subsequently carried-out metallurgical examinations (cf. Figure 9b), in which the dimension of the melt pools were determined, have shown that the areas of increased temperature visible in the thermography exceeded the width of the melt pool.

## 4. Discussion

The test series carried out in this research demonstrates the high precision that the synchronized galvanometer scanners can follow, even for complex paths. The brightest pixel selected as a benchmark provides a reliable reference point for the operating laser. The discrepancy between the strongly focused spot diameter and the resolution capability of the camera system provides a clear drop-off between the zone of energy input and the surrounding heat-affected area. In all combinations of power and traverse speed investigated, a single pixel always stood out clearly, fitting the characteristic properties of the laser melting process of local extremely high temperatures and a rapid drop close beside it. 

The accuracy of the setup reaches or exceeds the resolution capability of the camera system. Across all tests, oscillating deviations during the movement were not observed. The shift within the field of view, nevertheless occurring with high repeatability, can clearly be attributed to a distortion of the optical path that was not completely equalized for the observed wavelengths.

The initial results of this study illustrate the combination of the benefits of on- and off-axis monitoring within the SPIT approach. The system allows the use of wavelength ranges in the SWIR that were previously described by Mohr [20] as favorable for observations in the PBF LB/M process. In contrast with off-axis systems, there is no need to compromise between resolution, frame rate and observable working space [7]. Furthermore, due to the characteristics of the data acquisition consisting of a small movable measuring field, the SPIT setup enables high frame rates and resolutions with a simultaneously large observable working space. These attributes are particularly characteristic of on-axis systems [26]. In addition, it is possible to design the travel paths of the two independent galvanometer scanners differently, in principle.

By utilizing metallurgical investigations, the dimensions of the generated melt pools could be mapped onto corresponding infrared images. It was noticeable that the heated zone detected by the camera was significantly larger than the actual melt pool. This indicates that even heated areas that have not been melted can be captured in detail with the SPIT setup, and subsequently evaluated. 

## 5. Conclusions

In this paper, the SPIT setup was introduced as a novel monitoring system that enables high-resolution observation of the melt pool and the surrounding heat-affected zone in the PBF-LB/M process. The use of two independent optical paths permitted the usage of wavelength ranges favorable for thermography.

Initial trials with a variety of simple and complex laser paths approved the repeatability, with very high precision of two synchronized galvanometer scanners, even at high scan speeds. Furthermore, the point of interaction between the laser and the aluminum was clearly recognizable as a single pixel with prominent brightness. The expansion of the melt pool was limited to a very small spot in the field of view and surrounded by a significant area of thermal influence, which was dependent on the test setup for laser energy and travel speed. 

In the next steps, dedicated upgrades in the optical path will take place. The currently used 50 mm lens will be upgraded to an InfraTec telephoto lens with a focal length of 100 mm. Preliminary experiments with this advanced setup have shown a significant improvement in the achievable resolution without a significant loss of signal strength.

In order to enable more control of incident radiation to reduce the influence of interferences and allow more precise temperature mapping, a narrow band-pass filter will be installed into the sensor path. Tests already carried out have shown that an additional loss of signal power remains within manageable limits.

The observed shifts of the laser spot within the field of view caused by the optical refraction showed that a customized correction factor to equalize the movement completely is necessary. This factor will be implemented after the conduction of additional trails to map the currently existing distortion.

For future measurements of absolute temperatures, there will be investigations into moving the focal point apart from the zone of direct laser interaction and towards more stable areas regarding the aggregate state of the matter. Therefore, a predefined delay of the sensor path in regard to the laser will be applied. This disengagement of the momentarily linked scanners will be achieved via a custom software solution with a customizable gap between laser and sensor focus.

The goal of an obtainment of absolute temperatures requires accurate calibration of the camera, including the entire optical path with all its elements. 

Following these steps, the presented setup consisting of a high-speed SWIR camera and two synchronized galvanometer scanners will enable the measurement of absolute temperatures and temperature gradients in a defined region around the laser–material interaction point in great detail. The information gained will then be used as indicators to register anomalies occurring during manufacturing, to evaluate the process stability and predict the achieved part quality.

## Figures and Tables

**Table 1:** Laser energy outputs and travel speeds.

Energy Output	Travel Speed
105 W	0.05 m/s
240 W	0.5 m/s
460 W	1.0 m/s

## Data Availability

The data presented in this study are available on request from the corresponding author.

## References

[1] Baumers M., Carmignato S., Leach R. (2020). Introduction to Precision Metal Additive Manufacturing. Precision Metal Additive Manufacturing.

[2] Kempen K., Yasa E., Thijs L., Kruth J.-P., Van Humbeeck J. (2011). Microstructure and Mechanical Properties of Selective Laser Melted 18Ni-300 Steel. Phys. Procedia.

[3] Grasso M.L.G., Remani A., Dickins A., Colosimo B.M., Leach R.K. (2021). In-Situ Measurement and Monitoring Methods for Metal Powder Bed Fusion–An Updated Review. Meas. Sci. Technol..

[4] Trapp J., Rubenchik A.M., Guss G., Matthews M.J. (2017). In Situ Absorptivity Measurements of Metallic Powders during Laser Powder-Bed Fusion Additive Manufacturing. Appl. Mater. Today.

[5] Wehnert K.K., Ochs D., Schmitt J., Hartmann J., Schiffler A. (2021). Reducing Lifecycle Costs Due to Profile Scanning of the Powder Bed in Metal Printing. Procedia CIRP.

[6] Ali U., Mahmoodkhani Y., Shahabad S.I., Esmaeilizadeh R., Liravi F., Sheydaeian E., Huang K.Y., Marzbanrad E., Vlasea M., Toyserkani E. (2018). On the Measurement of Relative Powder-Bed Compaction Density in Powder-Bed Additive Manufacturing Processes. Mater. Des..

[7] Zhang Y., Fuh J.Y.H., Ye D., Hong G.S. (2019). In-Situ Monitoring of Laser-Based PBF via off-Axis Vision and Image Processing Approaches. Addit. Manuf..

[8] Mohr G. Measurement of Particle Emissions in Laser Powder Bed Fusion (L-PBF) Processes and Its Potential for In-Situ Process Monitoring. Proceedings of the Euro PM 2019.

[9] Dunbar A.J., Denlinger E.R., Heigel J., Michaleris P., Guerrier P., Martukanitz R., Simpson T.W. (2016). Development of Experimental Method for in Situ Distortion and Temperature Measurements during the Laser Powder Bed Fusion Additive Manufacturing Process. Addit. Manuf..

[10] Kleszczynski S., zur Jacobsmühlen J., Reinarz B., Sehrt J.T., Witt G., Merhof D. Improving Process Stability of Laser Beam Melting Systems. Proceedings of the Fraunhofer Direct Digital Manufacturing Conference.

[11] Schmidt M., Merklein M., Bourell D., Dimitrov D., Hausotte T., Wegener K., Overmeyer L., Vollertsen F., Levy G.N. (2017). Laser Based Additive Manufacturing in Industry and Academia. Cirp Ann..

[12] Mani M., Feng S., Lane B., Donmez A., Moylan S., Fesperman R. (2015). Measurement Science Needs for Real-Time Control of Additive Manufacturing Powder Bed Fusion Processes.

[13] Höfflin D., Rosilius M., Seitz P., Schiffler A., Hartmann J. (2022). Opto-Thermal Investigation of Additively Manufactured Steel Samples as a Function of the Hatch Distance. Sensors.

[14] Grasso M., Colosimo B.M. (2017). Process Defects and in Situ Monitoring Methods in Metal Powder Bed Fusion: A Review. Meas. Sci. Technol..

[15] Everton S.K., Hirsch M., Stravroulakis P., Leach R.K., Clare A.T. (2016). Review of In-Situ Process Monitoring and in-Situ Metrology for Metal Additive Manufacturing. Mater. Des..

[16] Krauss H. (2017). Qualitätssicherung Beim Laserstrahlschmelzen Durch Schichtweise Thermografische In-Process-Überwachung.

[17] Mohr G., Altenburg S.J., Ulbricht A., Heinrich P., Baum D., Maierhofer C., Hilgenberg K. (2020). In-Situ Defect Detection in Laser Powder Bed Fusion by Using Thermography and Optical Tomography—Comparison to Computed Tomography. Metals.

[18] Zenzinger G., Bamberg J., Ladewig A., Hess T., Henkel B., Satzger W. (2015). Process Monitoring of Additive Manufacturing by Using Optical Tomography. AIP Conf. Proc..

[19] Oster S., Maierhofer C., Mohr G., Hilgenberg K., Ulbricht A., Altenburg S.J. (2021). Investigation of the Thermal History of L-PBF Metal Parts by Feature Extraction from in-Situ SWIR Thermography. Proceedings of the Thermosense: Thermal Infrared Applications XLIII.

[20] Mohr G., Nowakowski S., Altenburg S.J., Maierhofer C., Hilgenberg K. (2020). Experimental Determination of the Emissivity of Powder Layers and Bulk Material in Laser Powder Bed Fusion Using Infrared Thermography and Thermocouples. Metals.

[21] Bartlett J.L., Heim F.M., Murty Y.V., Li X. (2018). In Situ Defect Detection in Selective Laser Melting via Full-Field Infrared Thermography. Addit. Manuf..

[22] Boone N. (2020). Near Infrared Thermal Imaging for Process Monitoring in Additive Manufacturing. Ph.D. Thesis.

[23] Bamberg J., Zenzinger G., Ladewig A. In-Process Control of Selective Laser Melting by Quantitative Optical Tomography. Proceedings of the 19th World Conference on Non-Destructive Testing.

[24] Craeghs T., Clijsters S., Kruth J.-P., Bechmann F., Ebert M.-C. (2012). Detection of Process Failures in Layerwise Laser Melting with Optical Process Monitoring. Phys. Procedia.

[25] Berumen S., Bechmann F., Lindner S., Kruth J.-P., Craeghs T. (2010). Quality Control of Laser-and Powder Bed-Based Additive Manufacturing (AM) Technologies. Phys. Procedia.

[26] Clijsters S., Craeghs T., Buls S., Kempen K., Kruth J.-P. (2014). In Situ Quality Control of the Selective Laser Melting Process Using a High-Speed, Real-Time Melt Pool Monitoring System. Int. J. Adv. Manuf. Technol..

[27] Harbig J., Wenzler D.L., Baehr S., Kick M.K., Merschroth H., Wimmer A., Weigold M., Zaeh M.F. (2022). Methodology to Determine Melt Pool Anomalies in Powder Bed Fusion of Metals Using a Laser Beam by Means of Process Monitoring and Sensor Data Fusion. Materials.

[28] Hartmann J. (2009). High-Temperature Measurement Techniques for the Application in Photometry, Radiometry and Thermometry. Phys. Rep..

[29] Wien W. (1894). Temperatur Und Entropie Der Strahlung. Ann. Phys..

[30] Ostermann F. (2014). Anwendungstechnologie Aluminium.

